# Does Preheating Resin Cements Affect Fracture Resistance of Lithium Disilicate and Zirconia Restorations?

**DOI:** 10.3390/ma14195603

**Published:** 2021-09-27

**Authors:** Amal Abdelsamad Sakrana, Walid Al-Zordk, Heba El-Sebaey, Ahmed Elsherbini, Mutlu Özcan

**Affiliations:** 1 Fixed Prosthodontics Department, Faculty of Dentistry, Mansoura University, Mansoura 35516, Egypt; walidwa@gmail.com (W.A.-Z.); h.elsebaey@yahoo.com (H.E.-S.); 2 Fixed Prosthodontics Department, Faculty of Dentistry, Horus University, Damietta 34511, Egypt; 3 Mansoura General Hospital, Mansoura 35511, Egypt; dr.ahmedelsherbini6@gmail.com; 4 Division of Dental Biomaterials, Center for Dental and Oral Medicine, Clinic for Reconstructive Dentistry, University of Zurich, 8032 Zurich, Switzerland; mutluozcan@hotmail.com

**Keywords:** pre-heating, adhesive cement, fracture resistance, IPS e.max Press, Blu-Zirkon

## Abstract

This paper assesses the impact of preheating of adhesive cement on the fracture resistance of lithium disilicate and zirconia restorations. Methods: A total of 80 human maxillary premolar teeth were assigned into 8 groups (*n* = 10) according to material type (either lithium disilicate or zirconia) and type of resin cement (either LinkForce or Panavia SA) with preheating temperature at 54 °C or at room temperature (25 °C). Teeth were prepared and restored with either lithium disilicate or zirconia restorations. After cementation, specimens were thermal cycled (10,000 cycles, 5 °C/55 °C), then load cycled for 240,000 cycles (50 N). Each specimen was statically loaded until fracture and the load (N) at fracture was recorded, then the failure mode was detected. Statistical analysis of data was performed (*p* ≤ 0.05). Results: There was no significant difference (*p* = 0.978) in fracture mean values between LinkForce and Panavia SA. Statistically significant difference (*p* = 0.001) was revealed between fracture resistance of lithium disilicate restorations cemented with LinkForce at 25 °C and at 54 °C; however there was no significant difference (*p* = 0.92) between the fracture resistance of lithium disilicate restorations cemented with Panavia SA used at 25 °C and at 54 °C. Regarding the interaction between ceramic material, cement type, and cement preheating, there was no significant effect (*p* > 0.05) in fracture resistance. The cement type does not influence the fracture resistance of ceramic restorations. Preheating of resin cement has negatively influenced the fracture resistance of all tested groups, except for lithium disilicate cemented using LinkForce cement.

## 1. Introduction

The main purpose of a luting agent is to fill the space between the prepared tooth and the restoration and to improve the structural durability of the restored tooth [1,2]. The appropriate selection and handling of luting agents is critical for success of indirect restorations [2]. Resin cements, with the capability of strong bond formation between the intaglio surface of the restoration and the prepared tooth structure, can offer benefits of reduced solubility with enhanced esthetics [3,4]. Therefore, resin cements could be used in challenging clinical situations of compromised retention or when low-strength ceramic restorations (such as glass-ceramics) are required [4,5]. Resin cements could seal the micro-cracks at the surface of the restoration and strengthen the restoration during function [6]. The thickness and type of a ceramic restoration could affect the polymerization of dual cure resin cement [7]. Self-adhesive resin cements are formulated to include adhesive polymers with the ability to form bond with the tooth structure with separate steps of etching, drying, and priming [8]. Self-adhesive resin cements were developed to simplify the adhesive bonding steps, save time, and reduce postoperative sensitivity with promising clinical outcomes [8,9,10].

Clinical application of ceramic restoration offers several advantages such as biocompatibility, excellent esthetic outcomes, and good mechanical properties including high fracture toughness and flexural strength [11,12,13,14,15,16]. Lithium disilicate ceramics have approximately 70 vol% of crystalline phase incorporated in the glassy matrix and have flexural strength about 360 MPa [17,18]. The heat-pressed lithium disilicate ceramics (IPS e.max Press, Ivoclar, Vivadent, Schaan, Liechtenstein) have higher strength and pleasing esthetic properties [19]. Zirconia based materials have the highest strength, fracture resistance, fracture toughness, and Vickers hardness among all other dental ceramic materials [20]. The main factors affecting the zirconia aging are the stabilizing oxide and its content, the grain size and the residual stress [21]. The most appropriate stabilizer is yttrium oxide (Y_2_O_3_) when added between 3.5 and 8 mol% [21]. Monolithic zirconia had proved to be a good esthetic restorative material [22]. The monolithic multilayer zirconia has been developed as a polychromatic, translucent material with combined shade and translucency gradient [23].

Prior to resin polymerization, composite resin preheating (up to 60 °C) reduces resin viscosity and enhances free radical mobility [24,25,26]. As a result, greater monomer conversion could be obtained at high temperatures than at room temperatures. Temperature affects polymerization kinetics as well as mechanical characteristics such as fracture resistance, shear strength, surface roughness and abrasion resistance [25].

The ultimate stress required to produce fracture or plastic deformation is described as fracture resistance, and it is significantly influenced by the magnitude of flaws and defects present on the surface of the tested materials [6,27,28,29]. Composite resin pre-heating prior to resin polymerization decreases the cement film thickness [24]. Reduced cement film thickness has been shown to decrease lithium disilicate restoration fracture [30]. Furthermore, it was found that increasing the cement layer thickness enhanced the fracture resistance of 1-mm ceramic plates [31]. The aim of this study was to assess the effect of preheating of adhesive and self-adhesive resin cements on fracture resistance of lithium disilicate and zirconia restorations. The first null hypothesis of the current study was that the cement type will affect the fracture resistance of lithium disilicate and zirconia restorations. Additionally, the preheating temperature of the resin cement will affect the fracture resistance of lithium disilicate and zirconia restorations.

## 2. Materials and Methods

This study followed all guidelines by the Local Research Ethics Committee and received approval no. A10120219. It used freshly extracted human maxillary first premolars for periodontal or orthodontic reason and collected from the Oral and Maxillofacial Surgery Department, Faculty of Dentistry, Mansoura University, Egypt. The teeth were selected with homogenous dimensions and morphology. Cracked, carious, or restored teeth were excluded. All selected teeth were disinfected for one week using diluted sodium hypochlorite (Clorox Bleach, Clorox Egypt Co., Cairo, Egypt) for 20 min at room temperature, then thoroughly washed with water, and stored in standardized saline solution (Sodium Chloride BP 0.9%, Fibco, Alexandria, Egypt) at room temperature until use. A power analysis was made using a computer software (G*Power v3.0.10) to detect the proper sample size based on the results of the previous studies.

**Figure 1:**
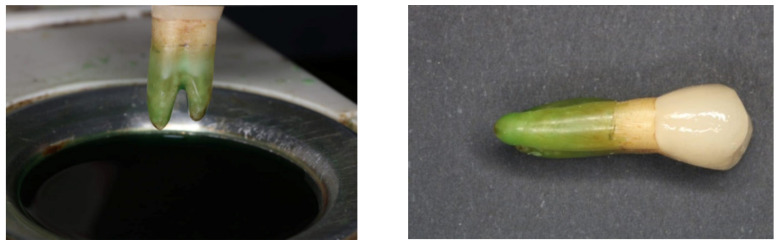
The root was covered with a thin layer of wax using the wax dipping technique.

The root of each tooth was dipped into a molten wax (3 mm away from cement–enamel junction) as shown in Figure 1. Each tooth was vertically mounted in an acrylic resin (Denture Base Polymers, Huge Dental Material Co., Shanghai, China). After setting, the tooth was removed from the acyclic resin block and the root was cleaned carefully using boiling water to remove the wax. Finally, a light body material (Ghenesyl light body, Lascod, Italy) was injected through a mixing tip attached to an auto-mix gun (Detax system II, Detax GmbH, Ettlingen, Germany) inside the mold of the acrylic block and the tooth was seated under pressure to simulate the periodontal ligament [13].

**Figure 2:**
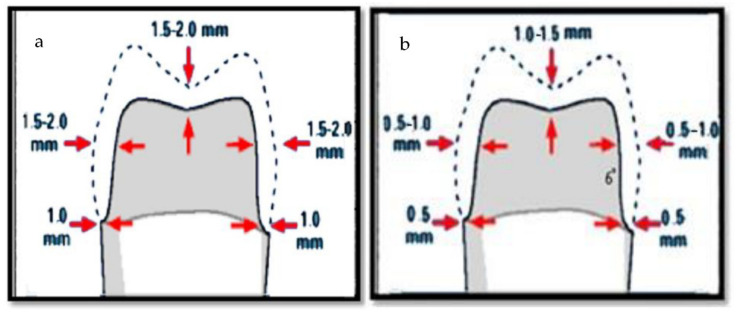
Illustration of tooth preparation to receive: (**a**) lithium disilicate restoration; (**b**) zirconia restoration.

An amount of 80 selected teeth were divided into 8 groups (*n* = 10) according to the restoration material type (zirconia or lithium disilicate), the cement type (Panavia SA cement plus or G-Cem LinkForce cement), and the preheating temperature at 54 °C or at 25 °C as shown in Table 1. For teeth preparation, the CAD/CAM system was used to standardize the preparation [32]. Standardization started with 2 teeth which were prepared using a hand piece attached to a dental surveyor (Dentalfarm A3006 B, Turin, Italy). For lithium disilicate, the preparation was 1 mm chamfer finish line, 2 mm functional cusp reduction, and 1.5 mm non-functional cusp reduction. For zirconia, the preparation was 0.5 mm chamfer, 1.5 mm functional cusp reduction, and 1 mm for non-functional cusp reduction as shown in Figure 2. The preparation was checked using a pre-preparation putty index (Imflex Putty, Meta Biomed Co., Chungcheongnam-do, Korea).

Fabrication of lithium disilicate restorations (*n* = 40): Each tooth was sprayed with anti-reflection scan powder spray (Telescan white, DFS Diamon GmbH, Riedenburg, The Netherlands) and scanned (ceramill Map 400, Amann Girrbach GmbH, Koblach, Austria), then the restorations were designed (Ceramill Mind, Amann Girrbach GmbH, Koblach, Austria). An 80 μm cement space was selected. The completed design was 3D printed (Phrozen shuffle, phrozen shuffle tech Co LTD, Hsinchu, Taiwan) with 3D printer resin (FTD Dentifix-3D LR, 3D printing resin, Lumi industries, Montebelluna, Italia). The spruing, investing, and pressing were performed following the manufacturer recommendations. After divesting, the restorations were cleaned and the sprues were cut. The external surfaces of the restorations were coated with the glaze (IPS e.max Ceram Glaze, Ivoclar Vivadent, Schaan, Liechtenstein), and restorations were subjected to a crystallization and glaze cycle following the manufacturer recommendations. For each restoration, the fitting surface was treated with 9% hydrofluoric acid gel (Porcelain etch, Ultradent, UT, USA) for 20 s.

Fabrication of zirconia restoration (*n* = 40): After scanning of the teeth and designing of the restorations, zirconia restorations were dry milled from zirconia block (Blu Zirkon tech 5D HT A2, Simex, Persiceto BO, Italy) using CAD-CAM milling machine (Ceramill motion II, Amann Girrbach GmbH, Koblach, Austria). The milled restorations were sintered (Ceramill Therm 3, Amann Girrbach GmbH, Koblach, Austria), then glazed based on the manufacturer recommendations. The fitting surface of each zirconia restoration was an air-borne particle abraded using 50 μm Al_2_O_3_ / 0.2 MPa (Renfert GmbH, Hilzingen, Germany).

Cementation: Panavia SA and LinkForce tubes were placed inside a digital incubator (Series BD model 56, Standard Incubators, BINDER GmbH, Tuttlingen, Germany) and adjusted according to the desired temperature required for cementation [33]. For LinkForce groups, the prepared teeth surfaces were treated with a brush saturated with the bonding agent (G-Premio Bond, GC Corp., Tokyo, Japan) for 15 s to evaporate the solvent, then cured using (LED curing light, Guilin Woodpecker Medical Instrument Co., Guangxi, China) for 20 s based on the manufacturer recommendation. LinkForce groups, the fitting surfaces of zirconia restorations, were treated with primer (G-Multi Primer, GC Corp., Tokyo Japan) for 10 s based on the manufacturer recommendation. For all groups, the corresponding resin cement was dispensed in the fitting surface of the restorations. Then, each restoration was seated on its corresponding tooth and held under constant load of 10 N (Instron Universal testing machine, 3345, MA, USA) during polymerization [34]. Each restoration was light cured for 3 s to allow removal of excess cement. Then, each surface was subjected to final curing for 20 s based on the manufacturer recommendations. After cementation, the specimens were stored in distilled water for 24 h.

Specimens were subjected to 10,000 cycles of thermal cycle using (Thermo-cycler SD Mechatronic GmbH, Munich, Germany) at temperature between 5 °C and 55 °C for 20 s at 10 s dwell time [35], followed by mechanical cyclic loading for 240,000 load cycles using a chewing simulation machine (chewing simulator CS4.4, SD Mechatronic GmbH, Munich, Germany) with a 50 N load at 60 mm/s.

At a crosshead speed of 0.5 mm/min, all specimens were subjected to a static compressive axial load using an instron universal testing machine (Model 3345, Instron, Canton, MA, USA). Tin foil of 0.5 mm thickness was placed over the occlusal surface of specimens to avoid contact damage. Each specimen was examined to determine the fracture mode. The fracture modes can be classified into: Class I: minimal fracture or crack in the crown; Class II: less than half of the crown is lost; Class III: half of the crown is lost; Class IV: more than half of the crown is lost; and Class V: severe fracture of the crown and/or the tooth [36]. Representative specimen of each fracture mode was selected and examined using a Scanning Electron Microscope. Significance of obtained results was judged at the (0.05) level. Multiple way ANOVA test was used for detection of combined effects on dependent outcome. Student’s *t*-test was used to compare two independent groups for parametric values.

## 3. Results

With neglecting the effect of resin cement type and its preheating, zirconia restorations showed statistically (t = 9.58, *p* = 0.001) higher fracture resistance compared with lithium disilicate restorations.

There was statistical significant difference (t = 4.64, *p* = 0.001) between the fracture resistance values of lithium disilicate restorations cemented with LinkForce cement at 25 °C (613.12 ± 119.65 N) and preheated at 54 °C (1015.39 ± 246.54 N); however, there was no statistical significant difference (t = 0.10, *p* = 0.92) between the fracture resistance values of lithium disilicate restorations cemented with Panavia SA cement at 25 °C (722.42 ± 125.52 N) and preheated at 54 °C (714.20 ± 229.01 N) (Table 2). Additionally, there was a statistically significant difference (t = 3.37, *p* = 0.004) between the fracture resistance values of zirconia restorations cemented with LinkForce cement at 25 °C (1572.05 ± 193.08 N) and preheated at 54 °C (1163.67 ± 344.02 N), and there was a statistically significant difference (t = 6.06, *p* ˂ 0.001) between the fracture resistance values zirconia restorations cemented with Panavia SA cement at 25 °C (1770.92 ± 270.65 N) and preheated at 54 °C (1168.03 ± 160.34 N). When comparing the fracture resistance of lithium disilicate restorations cemented with preheated cement, there was a statistically significant difference (t = 2.83, *p* = 0.01) between LinkForce and Panavia SA groups, also the fracture resistance of zirconia restorations cemented with preheated cement, there was a statistically significant difference (t = 13.35, *p* ˂ 0.001) between LinkForce and Panavia SA groups.

The three-way ANOVA test showed that the interaction between ceramic material, cement type and cement preheating had no significant effect (*p* ˃ 0.05) on the fracture resistance as shown in Table 3.

The modes of failure within studied groups were presented in Figure 3 and Figure 4. Representative SEM images for fractographic analysis of representative specimens were shown in Figure 5, Figure 6, Figure 7, Figure 8, Figure 9 and Figure 10.

## 4. Discussion

The aim of this study was to assess the effect of preheating of adhesive and self-adhesive resin cements on fracture resistance of lithium disilicate and zirconia restorations. The first null hypothesis that the cement type will affect the fracture resistance of lithium disilicate and zirconia restorations was rejected. Regarding the second null hypothesis that the preheating temperature of the resin cement will affect the fracture resistance of lithium disilicate and zirconia restorations was partially accepted because the fracture resistance of all studied groups, except lithium disilicate cemented with LinkForce groups, showed decrease with the use of preheated resin cement.

In the current study, monolithic zirconia was used because it has good esthetic properties and excellent mechanical properties [20,21,22]. Due to its unique mix of strength and esthetic qualities, lithium disilicate (IPS e.max Press) was also utilized [14,17,18,19]. Instead of stainless, epoxy resin, and composite resin dyes, which do not replicate the true force distribution that occurs on crowns cemented to human teeth, this study used human extracted teeth [11]. Dentin, on the other hand, has a lower modulus of elasticity than stainless steel, and as a result, the inner crown surface experiences more shear stress when the tooth is deformed [12]. The teeth were mounted in acrylic resin, with a silicon light body that simulated periodontal ligaments because the periodontal ligament is a crucial component for stress distribution caused by the application of force to teeth [13]. Soft tissue has a non-linear and viscous mechanical reaction to external stress, which is similar to the properties of elastomeric materials utilized in impression procedures [13].

The CAD/CAM technology was used to prepare the teeth to ensure standardization of all prepared teeth. In this study, circumferential 1 mm chamfer finish line was used for lithium disilicate and circumferential 0.5 mm chamfer finish line for zirconia restorations with 6-degree taper was used. Different axial tapering angles were used for all ceramic preparation ranging from 4–12 degrees. However, a 6 degree tapered angle was the most commonly used [11,37,38]. In this study, thickness of CAD/CAM restorations was easily standardized during the designing phase of the restoration. According to manufacturer recommendation, minimal occlusal thickness of 1.5 to 2 mm is critical for ceramic restorations to withstand the masticatory forces, and 1.5 mm for the axial walls thickness was considered enough to mask the underlying dentin and/or the filling materials [37,39].

In this investigation, the means fracture resistance values of the zirconia group were higher than for lithium disilicate. These findings were in agreement with studies reported that monolithic zirconia crown showed higher resistance to fracture than monolithic IPS e.max Press crown [25,26]. The finer grain size of monolithic zirconia crowns, along with the tetragonal-monoclinic transformation toughening process, resulted in compressive stresses in the material, resulting in decreased crack propagation and, as a consequence, enhanced fracture resistance of zirconia crowns [16,28].

The current findings reveal that the means of fracture resistance values between Panavia SA and LinkForce groups were not significantly different. These findings agreed with a previous study that found no significant differences in the fracture resistance of Panavia and RelyX Unicem cements [29]. Furthermore, according to a study, there were no significant variations in the fracture resistance of Panavia and RelyX Unicem cements [30]. In this study, adhesive and self-adhesive resin cements were employed since they offered a simple and non-sensitive approach that did not require a separate etching process, time saving, and a significant reduction in the risk of post-operative sensitivity [10,40]. In general, resin cements play a significant role in crown fracture during loading by sealing micro-cracks on the material surface, reducing flaws, and increasing the energy required for crown catastrophic fracture [6].

In the current study, preheating of resin cements decreased the fracture resistance of all groups, except lithium disilicate restorations cemented using the LinkForce cement group. A previous study reported that composite resin preheating prior to resin polymerization decreased film thickness [24]. Lithium disilicate fracture could be minimized by reducing resin film thickness [31]. Another study found that increasing the cement thickness enhanced the fracture resistance of 1 mm ceramic plates [32]. Increased fracture resistance with lithium disilicate cemented with LinkForce could be attributed to the fact that dual cure resin cement polymerization is affected by the thickness and type of the ceramic, and lithium disilicate (IPS e.max Press) has greater translucency and light transmission, allowing for more polymerization [7]. Nano-indentation tests, on several self-etch adhesive systems, have also shown that, when handled appropriately, two-step self-etch adhesive may surpass one-step self-etch adhesive [10]. To the best of our knowledge, no published study tested the effect of preheating of resin cement on the fracture resistance of different ceramic restorations.

In the current investigation, the main mode of failure, demonstrated with stereoscopic and SEM images, was the fracture of the restoration and the tooth which can be attributed to the strong bonding of the restoration and the inherited strength of the tested ceramics.

As a limitation, only two types of ceramic materials with two types of adhesive cements were used in the current study. The study was performed to simulate limited period in oral environment. Regardless of the efforts to standardize the selection of teeth, variations still exist since it is difficult to select an optimum match for all selected teeth.

## 5. Conclusions

The following conclusions were obtained based on the findings of this in vitro investigation:

The tested zirconia restoration (Blu-Zirkon) has higher fracture resistance compared with lithium disilicate (IPS e.max Press) restoration. The cement type does not influence the fracture resistance of monolithic ceramic restorations. Preheating of resin cement has a negative influence on the fracture resistance of all tested groups, except lithium disilicate restorations cemented using the LinkForce cement group.

## Figures and Tables

**Figure 3:**
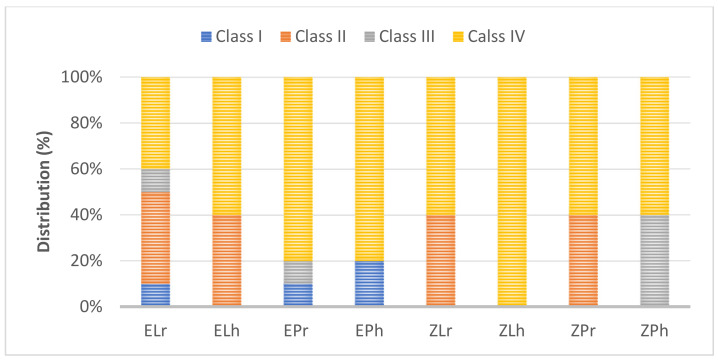
The mode of failure percentage (%) within study groups. Class I: Minimal fracture or crack in the restoration, Class II: Less than half of the restoration was lost, Class III: Half or more of the restoration was lost, and Class IV: Severe fracture of the restoration and/or the tooth.

**Figure 4:**
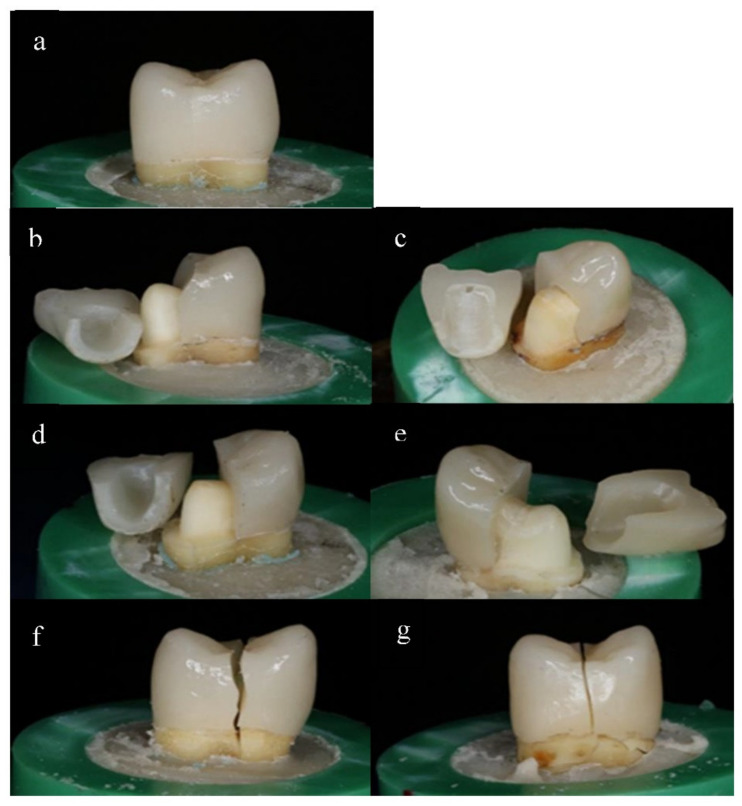
Stereomicroscopic images of the failure modes: (**a**) cracked lithium disilicate restoration cemented with Panavia SA at 25 °C (Class I); (**b**) fractured lithium disilicate restoration cemented with LinkForce at 54 °C (Class II); (**c**) fractured zirconia restoration cemented with Panavia SA at 25 °C (class II); (**d**) fractured lithium disilicate restoration cemented with Panavia SA at 25 °C (Class III); (**e**) fractured zirconia restoration cemented with Panavia SA at 54 °C (class III); (**f**) severe facture involving the lithium disilicate restoration and the tooth (class IV); (**g**) severe facture involving the zirconia restoration and the tooth (class IV).

**Figure 5:**
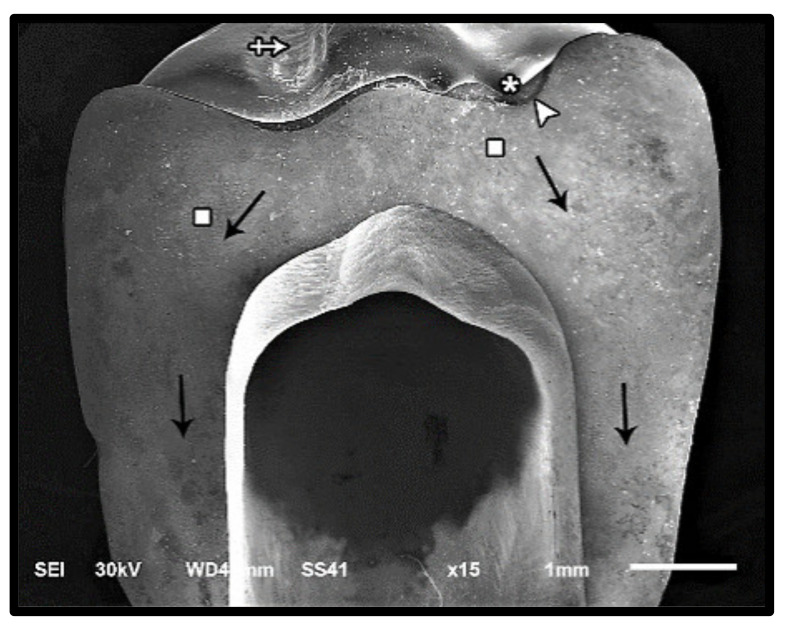
SEM micrograph represents class III failure mode of lithium disilicate fractured specimen showing the fracture origin (asterisk) at the loading occlusal surface of the restoration, hackle lines (square) indicating the direction of crack propagation (black arrow), arrested line (arrow head), and loss of glazing layer at the position of loading piston (cross-arrow).

**Figure 6:**
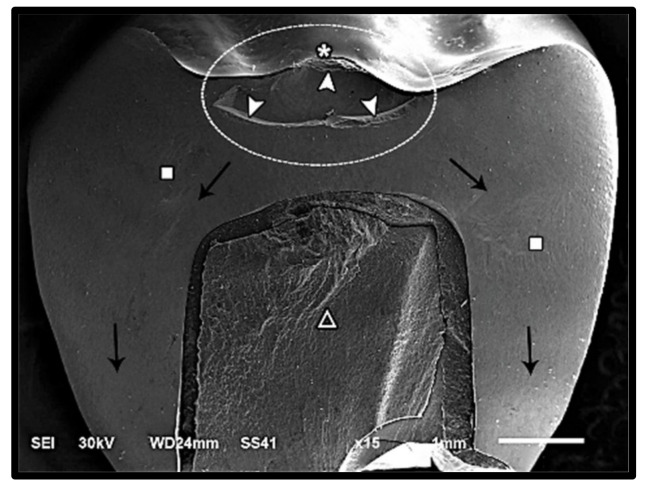
SEM micrograph represents class IV failure mode of lithium disilicate fractured specimen showing fracture origin (asterisk) at the loading occlusal surface of the restoration, hackle lines (square) indicating the direction of crack propagation (black arrow), arrest line (arrow-head) representing the limits of a small internal chip (dotted circle), and fracture of the restoration̸tooth complex (triangle).

**Figure 7:**
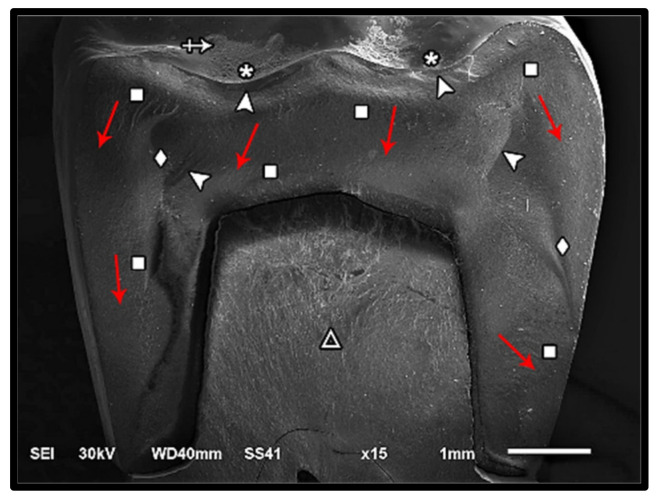
SEM micrograph for class III failure mode of zirconia fractured specimen showing fracture origins (asterisk) at the loading occlusal surface (two origins), hackle lines (square) indicating the direction of crack propagation from occlusal to cervical (red arrow), compression curl (diamond), diffused Arrest lines (arrow-head), loss of glazing layer at the position of loading piston (cross-arrow), and fracture of the restoration̸tooth complex (triangle).

**Figure 8:**
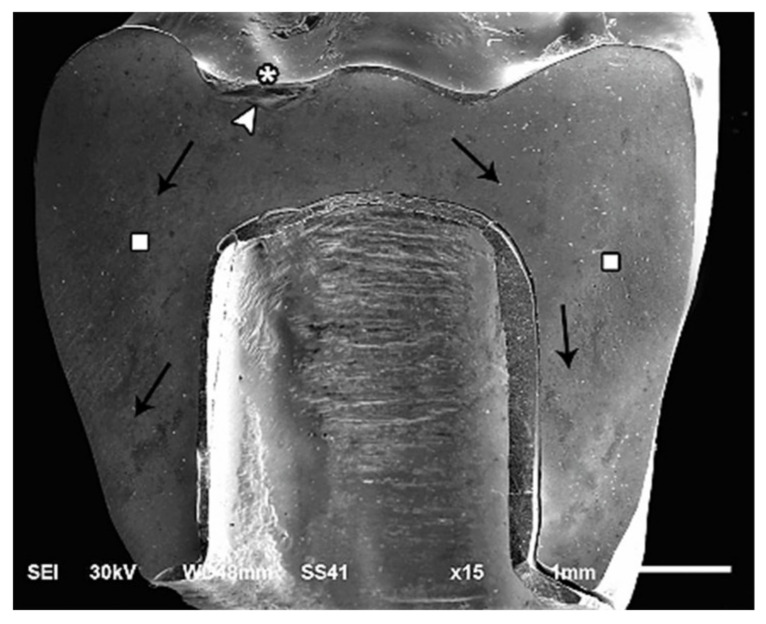
SEM micrograph represents class II failure mode of lithium disilicate fractured specimen showing fracture origins (asterisk) at the loading occlusal surface of the restoration and hackle lines (square) indicating the direction of crack propagation (black arrow), arrest line (arrow-head).

**Figure 9:**
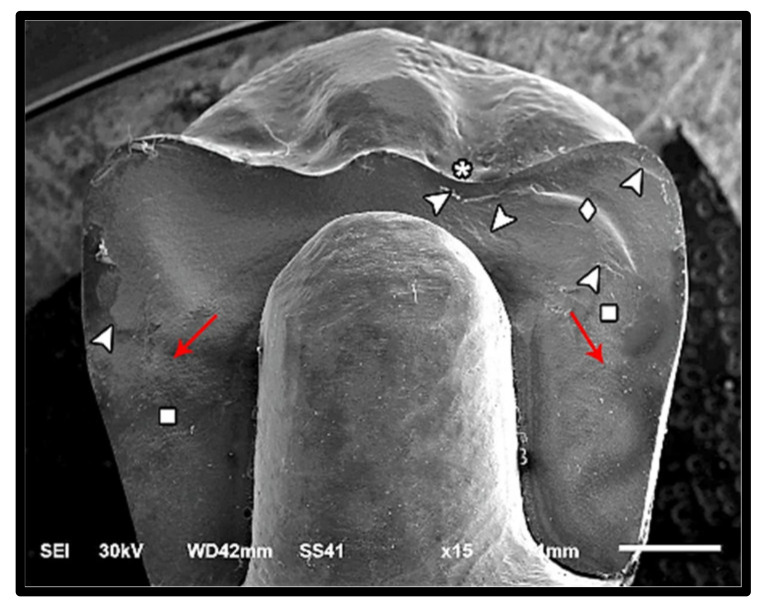
SEM micrograph represents class II failure mode of zirconia fractured specimen showing fracture origins (asterisk) at the loading occlusal surface, hackle lines (square) indicating the direction of crack propagation (red arrow), compression curl (diamond) indicating the crack propagation ends and begins in another direction before total fracture, and diffused arrest lines (arrow-head) through the fractured restoration.

**Figure 10:**
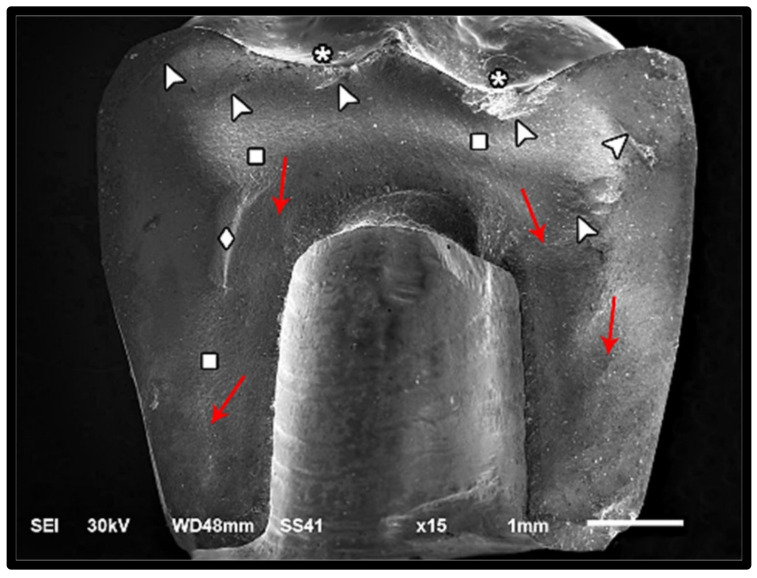
SEM micrograph represents class III failure mode of zirconia fractured specimen showing fracture origins (asterisk) at the loading occlusal surface of the restoration (two origins), hackle lines (square) indicating the direction of crack propagation from occlusal to cervical (red arrow), compression curl (diamond), and diffused arrest lines (arrow-head).

**Table 1:** The study groups.

Code	Group
ELr	Lithium disilicate restoration cemented using LinkForce at 25 °C
ELh	Lithium disilicate restoration cemented using LinkForce preheated at 54 °C
EPr	Lithium disilicate restoration cemented using Panavia S at 25 °C
EPh	Lithium disilicate restoration cemented using Panavia SA preheated at 54 °C
ZLr	Zirconia restoration cemented using LinkForce at 25 °C
ZLh	Zirconia restoration cemented using LinkForce preheated at 54 °C
ZPr	Zirconia restoration cemented using Panavia SA at 25 °C
ZPh	Zirconia restoration cemented using Panavia SA preheated at 54 °C

**Table 2:** Means and standard deviations of the fracture resistance (N) for studied groups.

Group	Mean ± SD
Lithium disilicate cemented with LinkForce at 25 °C	613.12 ± 119.65 ^A,B^
Lithium disilicate cemented with LinkForce at 54 °C	1015.39 ± 246.54 ^C,E^
Lithium disilicate cemented with Panavia SA at 25 °C	722.42 ± 125.52 ^A,D^
Lithium disilicate cemented with Panavia SA at 54 °C	714.20 ± 229.00 ^B,D^
Zirconia cemented with LinkForce at 25 °C	1572.05 ± 193.08
Zirconia cemented with LinkForce at 54 °C	1163.67 ± 344.02 ^C,F^
Zirconia cemented with Panavia SA at 25 °C	1770.92 ± 270.65
Zirconia cemented with Panavia SA at 54 °C	1168.03 ± 160.35 ^E,F^

Note: means with similar superscripted letters denote non-significant difference between groups within same column by Post Hoc Tukey test.

**Table 3:** Three-way ANOVA test regarding the fracture resistance (N) for the study groups.

	Type III Sum of Squares	Df	Mean Square	F	Sig.
Corrected Model	1.217 ^a^	7	1,738,313.375	34.951	0.000
Intercept	9.548	1	9.548	1.920	0.000
Materials	8,512,149.860	1	8,512,149.860	171.146	0.000
Temperature	476,209.919	1	476,209.919	9.575	0.003
Cement	160.688	1	160.688	0.003	0.955
Materials X Temperature	2,468,676.458	1	2,468,676.458	49.635	0.000
Materials X Cement	195,135.939	1	195,135.939	3.923	0.051
Temperature X Cement	457,549.400	1	457,549.400	9.200	0.003
Materials X Temperature X Cement	58,311.360	1	58,311.360	1.172	0.283
Error	3,581,014.608	72	49,736.314	-	-
Total	1.112	80	-	-	-
Corrected Total	1.575	79	-	-	-

^a^ R Squared = 0.751.

## Data Availability

The data presented in this study are available on request from the corresponding author A.A.S.
